# The Na^+/^H^+^-Exchanger NHE1 Regulates Extra- and Intracellular pH and Nimodipine-sensitive [Ca^2+^]_i_ in the Suprachiasmatic Nucleus

**DOI:** 10.1038/s41598-019-42872-w

**Published:** 2019-04-23

**Authors:** Pi-Cheng Cheng, Hsin-Yi Lin, Ya-Shuan Chen, Ruo-Ciao Cheng, Hung-Che Su, Rong-Chi Huang

**Affiliations:** 1 Department of Physiology and Pharmacology, College of Medicine, Chang Gung University, Tao-Yuan, 33302 Taiwan; 2 Healthy Aging Research Center, Chang Gung University, Tao-Yuan, 33302 Taiwan; 3 Neuroscience Research Center, Chang Gung Memorial Hospital, Linkou Medical Center, Tao-Yuan, 33305 Taiwan

**Keywords:** Circadian regulation, Cellular neuroscience, Neurophysiology

## Abstract

The central clock in the suprachiasmatic nucleus (SCN) has higher metabolic activity than extra-SCN areas in the anterior hypothalamus. Here we investigated whether the Na^+^/H^+^ exchanger (NHE) may regulate extracellular pH (pHe), intracellular pH (pHi) and [Ca^2+^]_i_ in the SCN. In hypothalamic slices bathed in HEPES-buffered solution a standing acidification of ~0.3 pH units was recorded with pH-sensitive microelectrodes in the SCN but not extra-SCN areas. The NHE blocker amiloride alkalinised the pHe. RT-PCR revealed mRNA for plasmalemmal-type NHE1, NHE4, and NHE5 isoforms, whereas the NHE1-specific antagonist cariporide alkalinised the pHe. Real-time PCR and western blotting failed to detect day-night variation in NHE1 mRNA and protein levels. Cariporide induced intracellular acidosis, increased basal [Ca^2+^]_i_, and decreased depolarisation-induced Ca^2+^ rise, with the latter two effects being abolished with nimodipine blocking the L-type Ca^2+^ channels. Immunofluorescent staining revealed high levels of punctate colocalisation of NHE1 with serotonin transporter (SERT) or CaV1.2, as well as triple staining of NHE1, CaV1.2, and SERT or the presynaptic marker Bassoon. Our results indicate that NHE1 actively extrudes H^+^ to regulate pHi and nimodipine-sensitive [Ca^2+^]_i_ in the soma, and along with CaV1.2 may also regulate presynaptic Ca^2+^ levels and, perhaps at least serotonergic, neurotransmission in the SCN.

## Introduction

The Na^+^/H^+^ exchanger (NHE) is an electroneutral (1:1 stoichiometry) antiporter that exchanges Na^+^ for H^+^ to regulate pH homeostasis in cytosol and organelles^1^. Nine genes are currently known to encode nine NHE isoforms (NHE1/SLC9A1–NHE9/SLC9A9) in the mammals^1–3^. The isoforms NHE1–5 are known as plasmalemmal-type as they are commonly found at the plasma membrane, whereas the isoforms NHE6–9 known as endomembrane-type as they are found in the organelles^1,3^. NHE1 is ubiquitously expressed with minimal basal activity in most tissues, but can be activated by intracellular H^+^ and is the principle mechanism for H^+^ extrusion in many cell types^1^. Importantly, the differential localisation of NHE1 along with other ion channels/transporters to distinct subregions of the plasma membrane allows it to regulate local pHi to influence many different cellular processes including membrane excitability, Ca^2+^ homeostasis, and neurotransmission^1,4,5^.

The hypothalamic suprachiasmatic nucleus (SCN) is the central clock controlling mammalian circadian rhythms of physiology and metabolism^6^. The SCN is metabolically more active during the day than at night, exhibiting a diurnal rhythm in glucose uptake^7,8^, cytochrome oxidase activity^9^, and Na/K pump activity^10^. On the other hand, the SCN is sensitive to metabolic perturbation and subject to regulation by metabolic cues such as the availability of glucose (see ref.^11^). While there is only limited knowledge available about how metabolic stress, such as glucose shortage, regulates the SCN, recent evidence indicates an important role of energy metabolism in the regulation of membrane excitability in the SCN neurones via the Na/K pump^12^ and the ATP-sensitive K^+^ channel^13^.

ATP hydrolysis during energy metabolism produces H^+^ ^14,15^, which may cause intracellular and extracellular acidification to impact H^+^ targets to regulate neuronal activity^16^. While steady pH gradient between the living tissue and the superfusate has been demonstrated in various neural tissues (see refs^16,17^), it is not known if this exists in the SCN. Such knowledge is particularly important, as we previously demonstrate that the SCN neurones are sensitive to mild extracellular acidification and express acid-sensing ion channels (ASIC), which contain high pH sensitivity of ASIC3 and ASIC1a subunits^18^. Furthermore, membrane conductances involved in neurotransmission such as NMDA receptors, GABA_A_ receptors, and voltage-gated calcium channels are also sensitive to intra- and extracellular protons^4,16^ and play a role in the regulation of circadian clock (see ref.^19^).

As the SCN is densely packed with neurones and has higher level of metabolic activity than extra-SCN areas^8^, we hypothesized that H^+^ produced during energy metabolism may be extruded by the NHE to influence both intracellular and extracellular pH in the SCN. We used ion-selective electrodes to measure the pHe values in hypothalamic slices containing the SCN, and ratiometric H^+^ and Ca^2+^ imaging to investigate the pHi and [Ca^2+^]_i_ in reduced SCN preparations. Real-time PCR and western blotting were used to investigate the NHE1 mRNA and protein levels, whereas immunostaining was used to investigate the distribution pattern and localisation of NHE1. Our results show that NHE1 actively extrudes H^+^ to cause extracellular acidification in hypothalamic SCN slices and maintain a more alkaline pHi to regulate [Ca^2+^]_i_ in the soma. Furthermore, double immunofluorescent staining revealed punctate colocalisation of NHE1 and CaV1.2 near the cell membrane, and triple staining of NHE1, CaV1.2, and serotonin transporter or the presynaptic marker Bassoon suggested that the NHE1 along with CaV1.2 may also regulate presynaptic Ca^2+^ levels and, perhaps at least serotonergic, neurotransmission in the SCN.

## Results

### Standing extracellular acidification in the SCN

**Figure 1:**
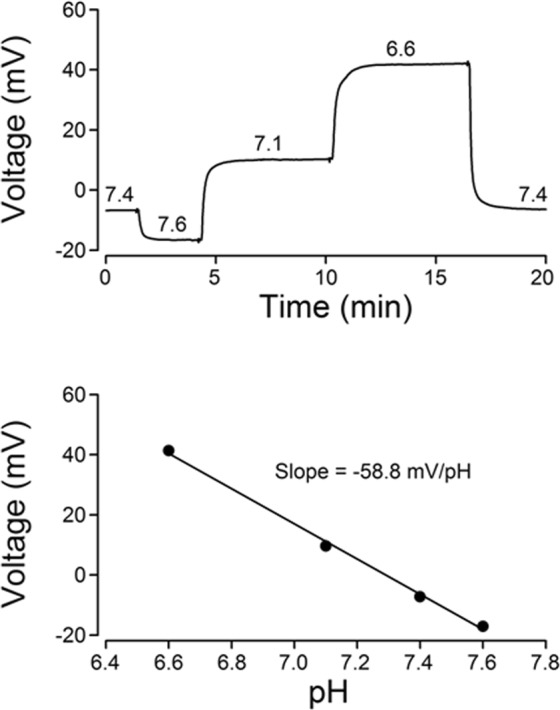
Calibration of a double-barreled pH-selective electrode. Top: Voltage responses to a series of solution change recorded with a double-barreled pH-selective microelectrode. The numbers on top of each voltage indicate the pH of each calibration solution (pH 6.6–7.6). Bottom: Liner regression plot of the calibration from the double-barreled pH-selective microelectrode.

**Figure 2:**
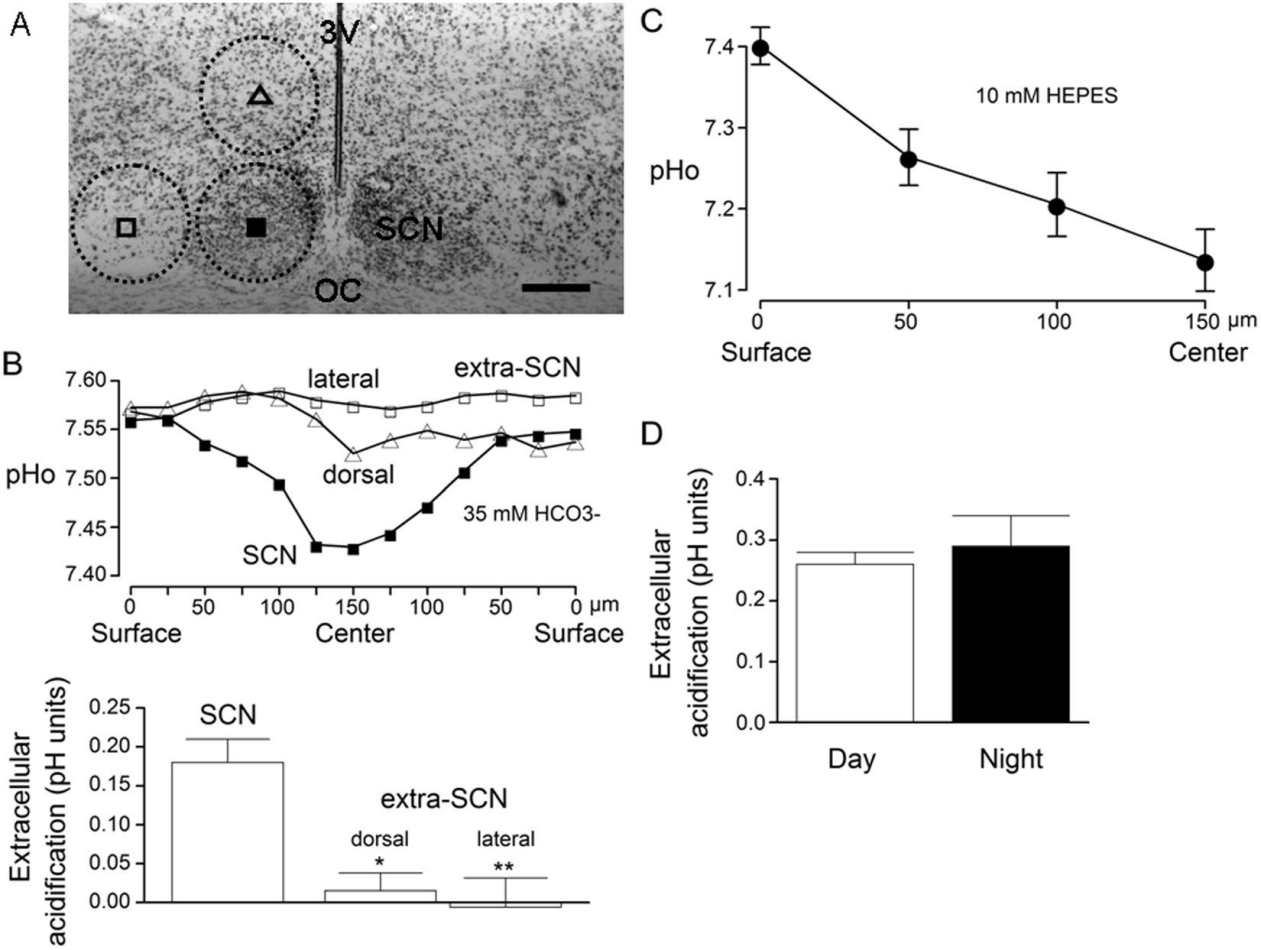
Extracellular pH measurements in the SCN and extra-SCN areas. (**A**) Nissl stain image showing the SCN and extra-SCN areas (encircled by broken lines). Symbols: approximate positions of double-barreled pH-sensitive electrodes. Scale bar: 200 µm. 3 V: third ventricle. OC: optic chiasm. (**B**) *Top*: Representative results obtained from a slice showing the extracellular pH measurements at different depths into and then out of the SCN (■) and the dorsal (Δ) and lateral (▢) extra-SCN region. The perfusion solution was buffered with 35 mM HCO_3_^−^/5% CO_2_ at pH 7.55. The slice thickness was 300 µm, and the surface was defined as 0 µm and the center as 150 µm. *Bottom*: Statistics comparing the average extracellular acidification in the center of the SCN and extra-SCN areas. *P < 0.05; **P < 0.01. (**C**) A standing pH gradient between the SCN and the perfusion solution buffered with 10 mM HEPES at pH 7.4. (D) Statistics showing a similar level of extracellular acidification recorded between day (ZT 4–11) and night (ZT 13–20).

The extracellular pH (pHe) of the rat SCN was measured with double-barreled pH-sensitive microelectrodes calibrated as described in Methods (Fig. 1). Figure 2A shows the Nissl stain image of the SCN and surrounding extra-SCN areas (encircled by broken lines), with the symbols indicating where the pH-sensitive microelectrode was positioned. To determine the steady pH gradients between the center in the slice (300 µm in thickness) and the bath, the electrode was first moved to the surface of the slice, and was then advanced every 25 or 50 µm into the center at a depth of 150 µm. Early experiments were performed to compare the pHe in the SCN and the surrounding extra-SCN regions in hypothalamic slices constantly perfused with a solution buffered with 35 mM HCO_3_^−^/5% CO_2_ (pH = 7.55). Figure 2B, top panel, shows the pHe values measured every 25 µm from the surface to the center and then back to the surface of the slice. Under conditions of 35 mM HCO_3_^−^/5% CO_2_, the pHe in the center of the SCN was ~7.40, an acidification of ~0.15 pH units, whereas the pHe in extra-SCN areas remained not much different from the bath of pH 7.55. On average the extracellular acidification (the difference in pH between the SCN and superfusion solution) in pH unit was 0.18 ± 0.03 (*n* = 8 slices) in the SCN, significantly larger (F_(2, 15)_ = 10.6, *P* = 0.0014, ANOVA) than the value of 0.015 ± 0.023 (*n* = 4 slices) and –0.006 ± 0.038 (*n* = 6 slices) in the dorsal and lateral extra-SCN areas, respectively (*bottom* panel).

When the slice was bathed in 10 mM HEPES-buffered (HCO_3_^−^ free) perfusion solution maintained at pH 7.4, the pHe in the center of the SCN was ~7.1, an acidification of ~0.3 pH units. Figure 2C shows the average pHe determined every 50 µm from the surface to the center of the SCN, the pH values being 7.40 ± 0.02 (*n* = 19 slices), 7.25 ± 0.04 (*n* = 17 slices), 7.19 ± 0.04 (*n* = 17 slices), and 7.12 ± 0.04 (*n* = 19 slices), respectively. On average, the extracellular acidification in the center of the SCN was 0.27 ± 0.02 pH units (*n* = 19 slices). Comparison of the extracellular acidification recorded between day (ZT 4–11) and night (ZT 13–20) indicates a similar degree of acidification, the values being 0.26 ± 0.02 pH units (*n* = 10 slices) and 0.29 ± 0.05 pH units (*n* = 9 slices) (t_(17)_ = 0.58, *P* = 0.58, unpaired *t*-test), respectively (Fig. 2D).

### Dose-dependent effects of amiloride on the SCN pHe

**Figure 3:**
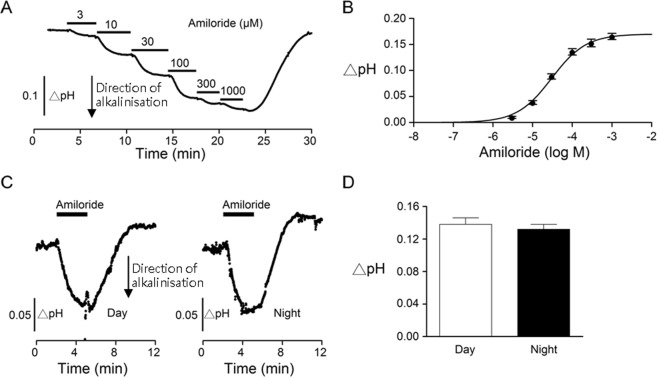
Amiloride effects on the extracellular pH (pHe) in the SCN in hypothalamic slices. (**A**) Dose-dependent effects of amiloride on the pHe in the SCN obtained from a representative experiment. (**B**) The dose-response relation was fitted with an equation assuming a one-to-one binding of amiloride to and blockade of the NHE. The fitted IC_50_ was 30 µM for amiloride binding to NHE and blockade of H^+^ extrusion. (**C**) Two representative experiments showing the pHe responses to 100 µM amiloride recorded at day (*left*) and at night (*right*). (**D**) Statistics showing a similar magnitude of amiloride-induced alkaline shifts between day and night.

The more acidic pHe in the SCN (than extra-SCN regions and superfusate) indicates continuing production^14,15^ and extrusion^16^ of protons from cells in the nucleus. To investigate the role of Na^+^/H^+^ exchanger (NHE) in extruding H^+^ to cause extracellular acidifications, the nonspecific blocker amiloride was applied to determine its effect on the pHe (Fig. 3). The result indicates a dose-dependent increase in the pHe by amiloride (Fig. 3A), suggesting an active role of NHE in mediating H^+^ efflux into extracellular space. The theoretic curve fitted to the dose-response relation yielded an IC_50_ of 30 µM, assuming a one-to-one binding and block of NHE (Fig. 3B).

To determine whether amiloride-induced alkalinisation varies between day and night, we compared the effect of 100 µM amiloride on the pHe (Fig. 3C,D). The result indicated a similar effect of 100 µM amiloride on the pHe between day (left panel) and night (right panel) (Fig. 3C). On average 100 µM amiloride-induced extracellular alkalinisation in pH unit was 0.14 ± 0.01 (*n* = 22 slices) during the day and 0.13 ± 0.01 (*n* = 11 slices) at night (t_(31)_ = 0.63, *P* = 0.53, unpaired *t*-test) (Fig. 3D).

### The SCN expresses the plasmalemmal-type NHE1, NHE4, and NHE5 isoforms

**Figure 4:**
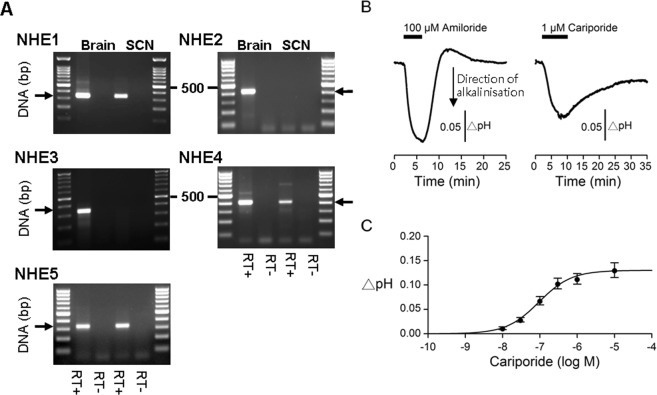
NHE1 is the major NHE isoform in mediating extracellular acid shifts in the SCN. (**A**) RT-PCR analysis of mRNAs for the plasmalemmal-type NHE isoforms in the SCN. Positive controls were performed using cDNA from rat brain. The expected PCR product sizes for NHE1–NHE5 were 324, 421, 306, 408, and 325 bp, respectively. Negative controls were performed using RT products with omission of reverse transcriptase (RT-) to examine the contamination of genomic DNA. (**B**) A representative experiment comparing the effects of 100 µM amiloride and 1 µM cariporide on the pHe. Note the slower kinetics of alkalinisation and re-acidification by cariporide than by amiloride. The reason for the slower kinetics is not known at present. (**C**) Summary of dose-dependent effect of cariporide on the pHe, with the dose-response relation fitted with an equation assuming a one-to-one binding of cariporide to and blockade of the NHE1. The fitted IC_50_ was 0.094 µM.

RT-PCR was used to determine the expression of the plasmalemmal-type NHE1–5 isoforms in the SCN (Fig. 4A). Positive control reactions were performed using cDNA of rat brain (NHE1–5) to determine the primer efficiency and anneal temperature. These primers were then used to examine the gene transcription of NHE1–5 isoforms in the SCN. The RT-PCR of SCN showed positive signals with primers of NHE1, NHE4, and NHE5 as compared with the RT- (with omission of reverse transcriptase) control (Fig. 4A). The result indicates the presence of mRNA for NHE1, NHE4, and NHE5 in the SCN.

### NHE1 is the major NHE isoform in mediating extracellular acid shifts in the SCN

The NHE1–5 isoforms are blocked by amiloride with different sensitivity, with a decreasing sensitivity in order of NHE1 > NHE5 > NHE4. Specifically, amiloride blocks the cloned rat NHE1 with an IC_50_ of 1.6 µM in one study^20^ and an IC_50_ of 5.3 µM and 813 µM, respectively, for NHE1 and NHE4, in another^21^, but an IC_50_ of ~1.5 µM and ~20 µM, respectively, for human NHE1 and NHE5^20,22^. An IC_50_ of 30 µM (determined with doses of amiloride up to 1 mM), as opposed to ~800 µM for amiloride block of NHE4, suggests a minimal, if any, contribution of NHE4 in mediating extracellular acidification in the SCN. On the other hand, although an IC_50_ of 30 µM is larger than those (1.5~5 µM) for cloned rat NHE1, the lack of information of amiloride sensitivity for rat NHE5 precluded us from making meaningful inference as to the participation of NHE5 in mediating standing extracellular acid shifts.

Nevertheless, the benzoylguanidines cariporide has been shown to inhibit NHE1 (IC_50_ ~0.03–3.4 µM) much more potent than NHE5 (IC_50_ > 30 µM)^23^ and at a concentration of 1 µM should have specifically inhibited NHE1. We thus compared the effect of 100 µM amiloride and 1 µM cariporide on the resting pHe from the same SCN slices (Fig. 4B). The result indicated a comparable change in the pHe, albeit with slower kinetics of pHe response to cariporide. On average, the peak pHe response to 100 µM amiloride and 1 µM cariporide averaged 0.17 ± 0.01 (*n* = 5 slices) and 0.16 ± 0.02 (*n* = 5 slices) (t_(4)_ = 0.72, *P* = 0.51, paired *t*-test), respectively. The result revealed a similar magnitude of alkaline shift induced by 100 µM amiloride and 1 µM cariporide. Figure 4C shows the dose-dependent effect of cariporide on the pHe. Curve fitting to the dose-response relation yielded an IC_50_ of 0.094 µM for cariporide-induced alkalinisation. This value falls within the range of reported values of IC_50_ for NHE1 and is much lower than that (IC_50_ > 30 µM) for NHE5^23^. Together the results indicate that NHE1 is the major NHE isoform in mediating extracellular acidification in the SCN.

### Real-time PCR and western blot analysis of the NHE1 expression

**Figure 5:**
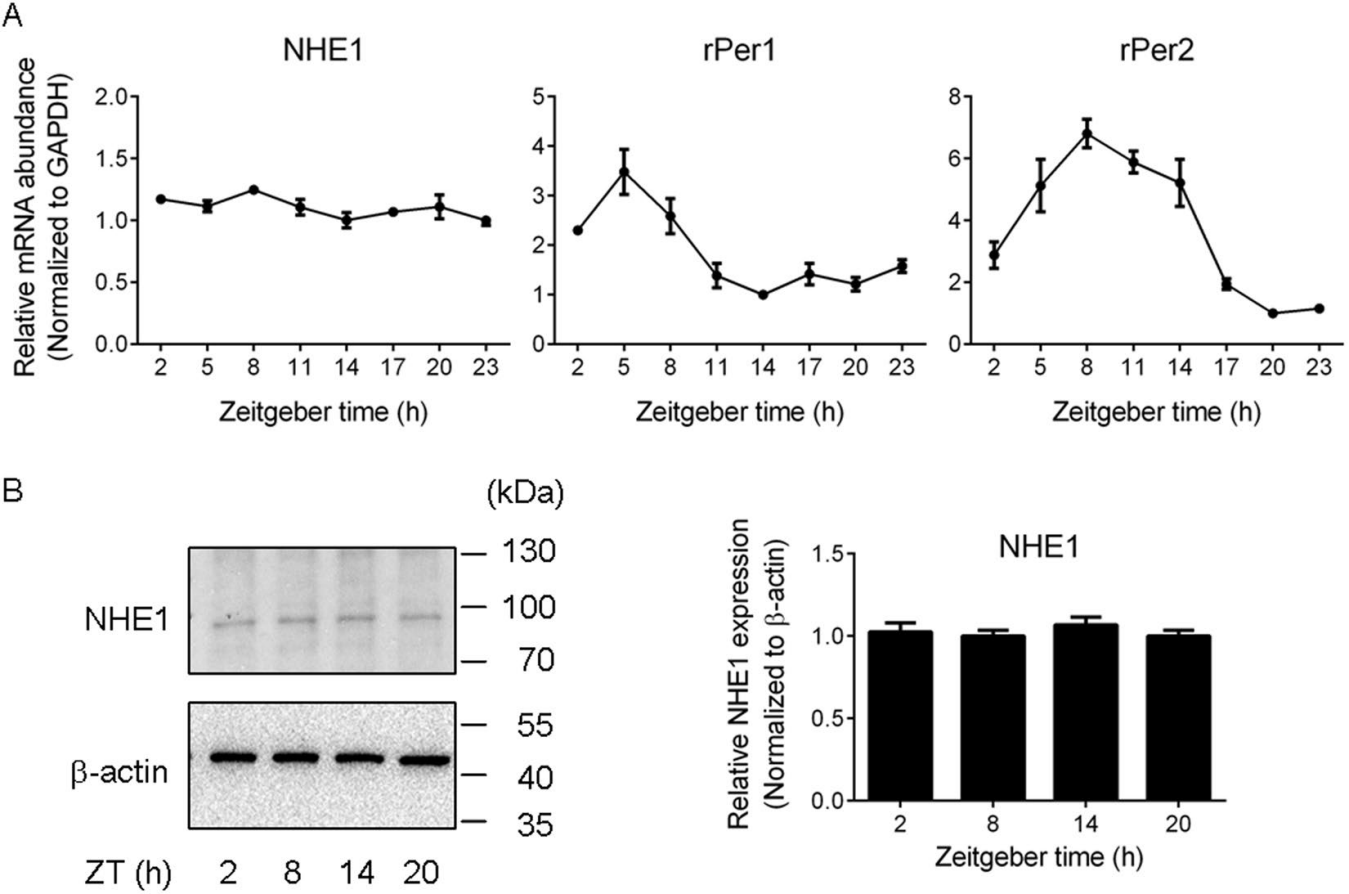
Daily profiles of NHE1 gene expression (**A**) and protein levels (**B**). (**A**) Real-time PCR results showing the daily profiles of gene expression for *NHE1* (*left*), *rPer1* (*middle*), and *rPer2* (*right*). (**B**) Western blot analysis showing the protein levels for NHE1 (~90 kDa; *left top*) and β-actin (~42 kDa; *left bottom*) at four different time points across the day. The full-length gels are presented in Supplementary Fig. 1. *Right*: Statistics showing similar expression levels of NHE1 among different time points.

To investigate whether the NHE1 expression is rhythmic in the SCN, we measured the mRNA and protein levels at different time points by real-time PCR and western blot analysis, respectively (Fig. 5). The real-time PCR failed to detect variation in the NHE1 gene expression across the time of the day (*n* = 4 per time point, total *n* = 32) (F_(7, 24)_ = 2.14, *P* = 0.08, ANOVA) (Fig. 5A, left panel). In contrast, the clock genes *rP*er1 (F_(7, 24)_ = 19.5, *P* < 0.0001, ANOVA) and *rP*er2 (F_(7, 24)_ = 23.2, *P* < 0.0001, ANOVA) both exhibited a robust rhythmicity, with the highest expression at ZT 5 and lowest at ZT 14 for *rPer1* (Fig. 5A, middle panel), and ZT 8 and ZT 20 for *rPer2* (Fig. 5A, right panel), similar to those reported previously in the rat SCN^24,25^. The western blot analysis also failed to detect day-night variation in the NHE1 protein levels (F_(3, 12)_ = 0.55, *P* = 0.66, ANOVA) (*n* = 4 per time points, total *n* = 16) (Fig. 5B).

### NHE1-mediated intracellular alkaline shifts

**Figure 6:**
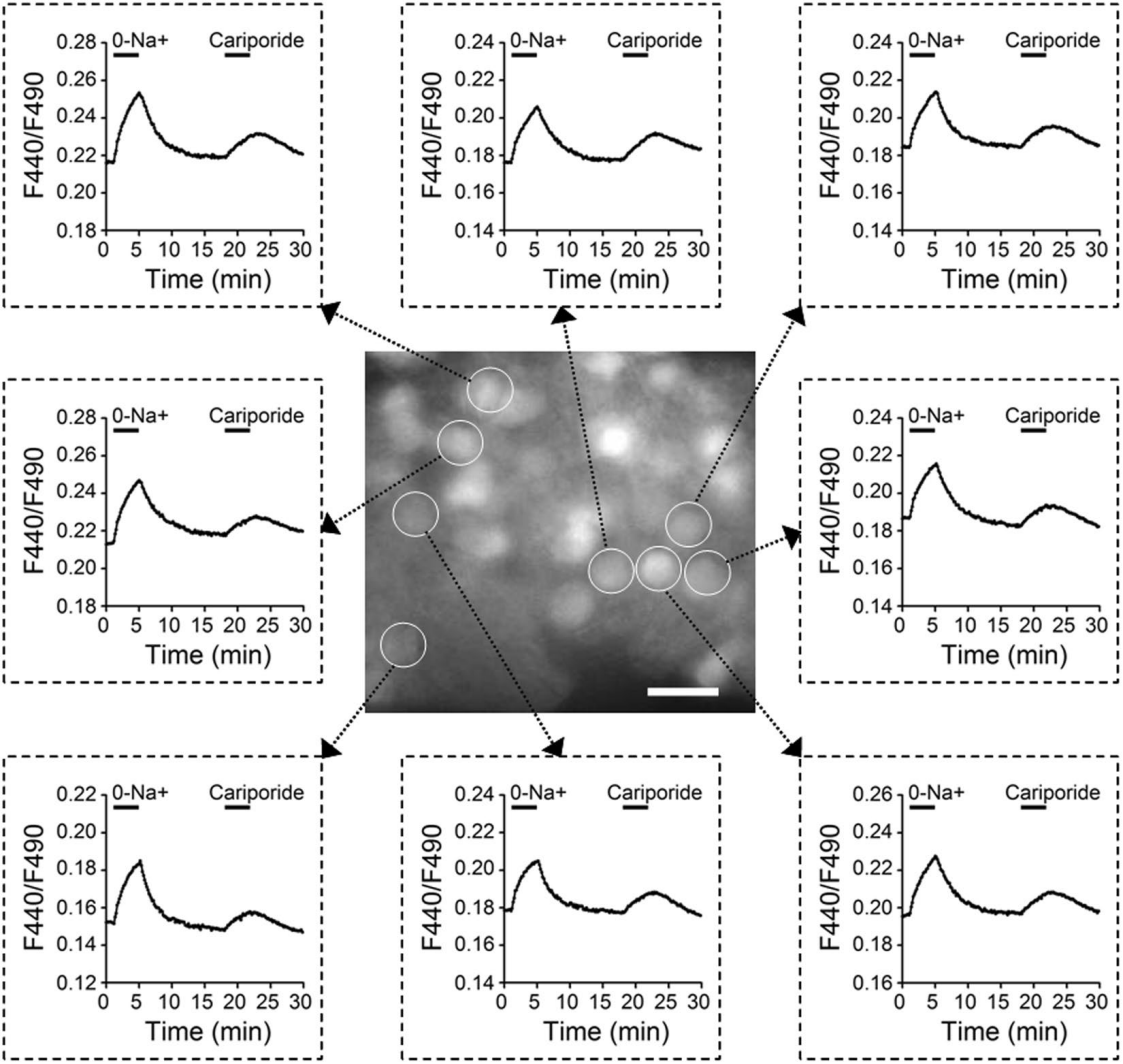
Effects of Na^+^-free solution and cariporide on the pHi in cells in reduced SCN slice preparations. *Center*, Fluorescence micrograph of a reduced SCN preparation loaded with the H^+^-sensitive fluorescent indicator BCECF-AM. Regions of interest (ROI) are indicated with circles. The image was taken in the resting condition. Scale bar: 20 µm. *Surround*, The time course of change in the F440/F490 fluorescence ratio recorded from 8 selected ROIs as indicated in *Center*.

The ability of cariporide to produce extracellular alkaline shifts suggests a constitutive activation of NHE1 in extruding H^+^ out of the cells to extracellular space in the SCN slice. In other words, NHE1 should help maintain intracellular pH (pHi) in more alkaline conditions. To test this idea, ratiometric proton imaging with BCECF-AM was used to measure the pHi change of cells in reduced SCN slice preparations. For the experiments, we investigated the effects of cariporide, as well as Na^+^-free solution, on baseline pHi (Fig. 6). The center picture shows part of a reduced SCN preparation, with selected cells circled to represent the ROI for averaging fluorescence signals. The surrounding plots of F440/F490 ratio indicate the change in [H^+^]_i_. In all circled cells, the blockade of NHE1 with 1 µM cariporide reversibly acidified the pHi (or increased [H^+^]_i_), suggesting a constitutive activation of NHE1 to extrude H^+^ to maintain a more alkaline pHi. Na^+^-free solution (0-Na^+^; NMDG^+^ substituted) also reversibly acidified the pHi, but to a larger extent than cariporide. A larger response by 0-Na^+^ was expected, because, in addition to inhibiting H^+^ extrusion via NHE1 as cariporide did, 0-Na^+^ could also increase intracellular H^+^ concentrations by promoting H^+^ uptake via reverse NHE1 activity (see, for example, ref.^26^).

### Cariporide effects on [Ca^2+^]_i_

**Figure 7:**
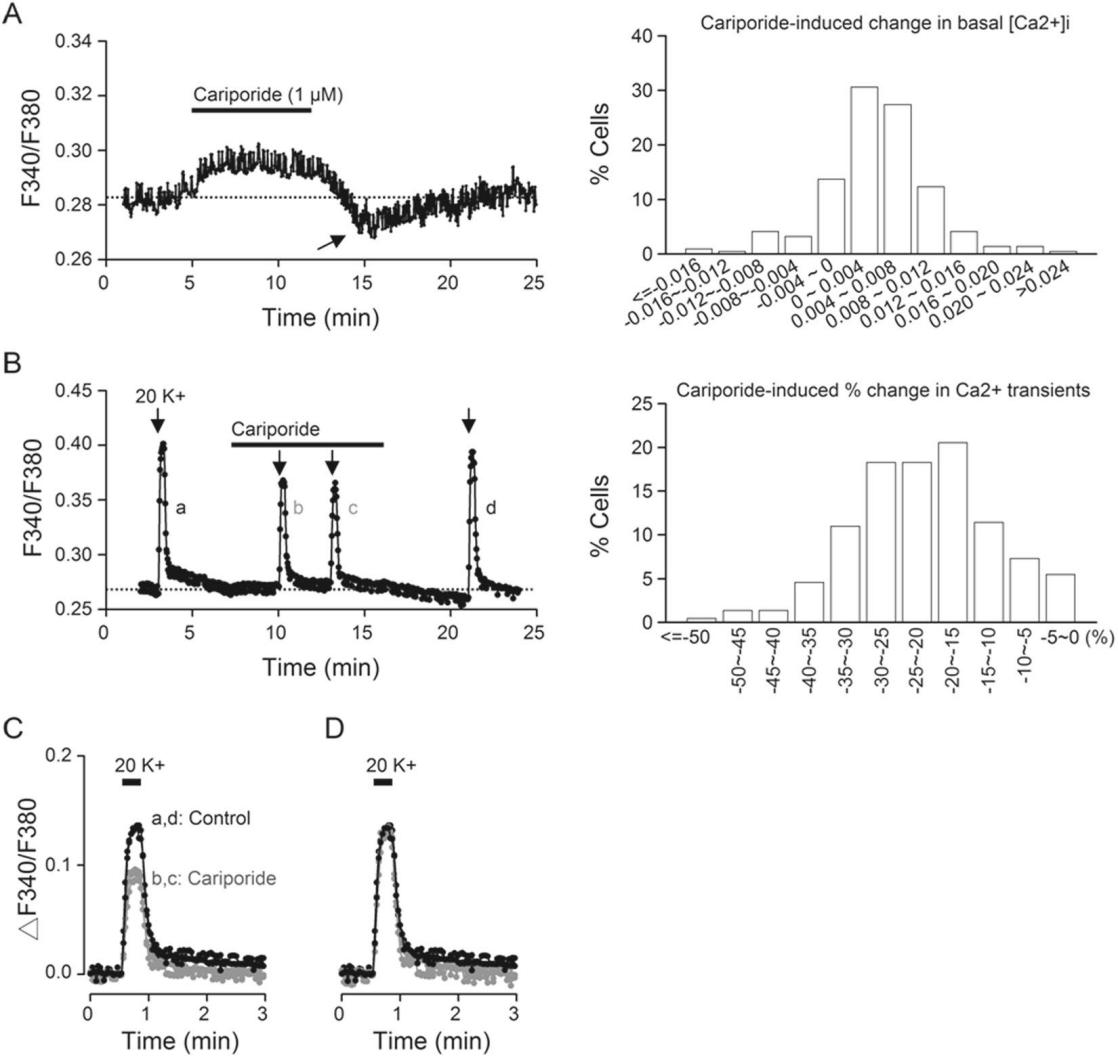
Cariporide effects on [Ca^2+^]_i_ in cells in reduced SCN slice preparations. (**A**) A representative experiment showing the effect of 1 µM cariporide on basal [Ca^2+^]_i_ (an average of 15 cells) (*left*). *Right*: Histogram showing the distribution of cariporide-induced changes in basal [Ca^2+^]_i_ (*n* = 219 cells from 11 experiments). (**B**) A different experiment to show the effect of 1 µM cariporide on 20 mM K^+^-induced Ca^2+^ rise (*left*). *Right*: Histogram showing the distribution of cariporide-induced percentage changes in Ca^2+^ transients (*n* = 219 cells). (**C**) Superimposition of the Ca^2+^ responses to 20 mM K^+^ before (*a*), during (*b*, *c*), and after (*d*) the application of cariporide. (**D**) Normalisation of Ca^2+^ transients to indicate the similarly fast decay time course.

Many proteins playing important roles in cellular physiology, including membrane excitability and Ca^2+^ handling, are highly sensitive to local pH change (for review, see ref.^27^). We previously reported that both the Na^+^/Ca^2+^ exchanger (NCX) and mitochondria play a role in the regulation of [Ca^2+^]_i_ in the rat SCN neurones, with NCX mediating fast Ca^2+^ decay following high K^+^-induced Ca^2+^ transients and mitochondria regulating basal [Ca^2+^]_i_^28,29^. Here we tested the idea that the constitutive activation of NHE1 may regulate [Ca^2+^]_i_ in the SCN neurones. We used ratiometric Ca^2+^ imaging to determine the effects of cariporide on basal [Ca^2+^]_i_ and Ca^2+^ rise in response to membrane depolarisation with 20 mM K^+^. Figure 7A, left panel, shows a representative Ca^2+^ response to the application of 1 µM cariporide, which increased basal [Ca^2+^]_i_ on application and then transiently lowered it to a level below control (marked by arrow) on washout (an average of 15 cells). Right panel shows the histogram for the distribution of cariporide-induced change in basal [Ca^2+^]_i_, with most cells showing elevated basal [Ca^2+^]_i_ in response to cariporide. On average, cariporide increases basal [Ca^2+^]_i_ by 0.0036 ± 0.0004 (*n* = 219 cells from 11 experiments).

In contrast to mostly increasing effect on basal [Ca^2+^]_i_, cariporide invariably inhibited the Ca^2+^ response to membrane depolarisation evoked by 20 mM K^+^ (Fig. 7B, left panel). Right panel shows the histogram for the distribution of cariporide-induced percent change of 20 (mM) K^+^-induced Ca^2+^ transients. On average, cariporide reduced peak Ca^2+^ transients by 22 ± 1% (*n* = 219 cells). Figure 7C superimposes the 20 K^+^-induced Ca^2+^ transients for better visualisation of the effect of cariporide. Normalisation of Ca^2+^ transients revealed similarly fast decay kinetics in the absence (dark traces) and the presence (grey traces) of cariporide (Fig. 7D), suggesting a negligible effect of cariporide on Ca^2+^ extrusion mediated by NCX1^28^. Together the results indicate that the blockade of NHE1 with cariporide increases basal [Ca^2+^]_i_ but inhibits the peak amplitude of depolarisation-evoked Ca^2+^ rise, without altering the fast decay kinetics.

### Acetate effects on [Ca^2+^]_i_

**Figure 8:**
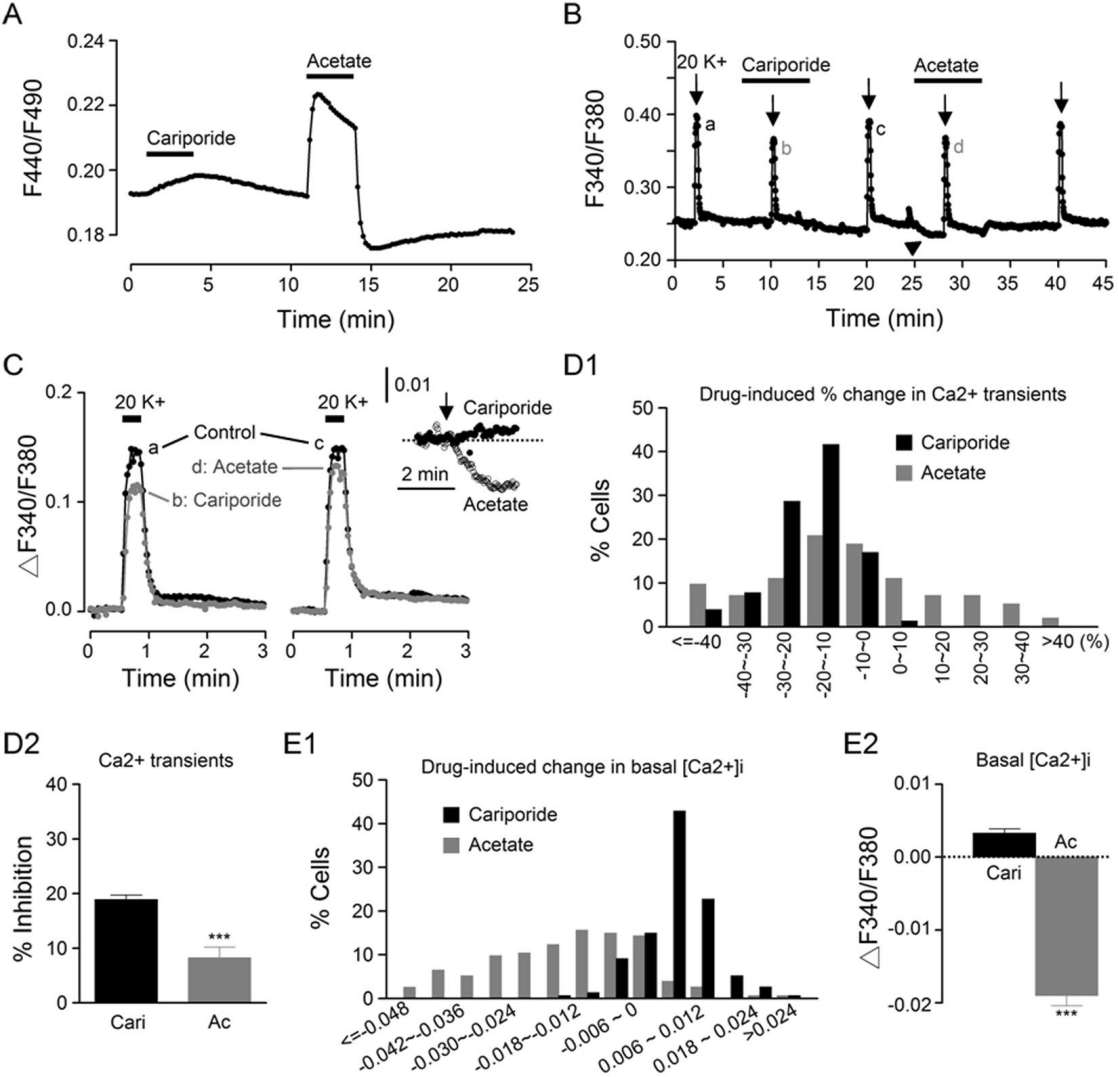
Effects of cariporide and sodium acetate on the pHi and [Ca^2+^]_i_ in the same SCN cells. (**A**) A representative experiment showing the effect of 1 µM cariporide and 20 mM sodium acetate on the pHi. (**B**) A representative experiment showing the effect of 1 µM cariporide and 20 mM sodium acetate on [Ca^2+^]_i_. Note the lowering effect of acetate on basal [Ca^2+^]_i_ (marked by arrowhead). **(C)** Superimposition of the Ca^2+^ transients to show the suppressive effect of cariporide (*left*) and acetate (*right*). *Inset*: Superimposition of basal [Ca^2+^]_i_ to indicate the opposite effects of cariporide (filled circles) and acetate (open circles). **(D1)** Histogram showing the distribution of percentage changes in Ca^2+^ transients produced by cariporide (black bars) and acetate (grey bars) (*n* = 154 cells from 8 experiments). **(D2)** Statistics showing the averaged inhibition by cariporide (Cari) and acetate (Ac) of the peak amplitude of 20 K^+^-induced Ca^2+^ rise. **(E1)** Histogram showing the distribution of changes in basal [Ca^2+^]_i_ produced by cariporide (black bars) and acetate (grey bars). **(E2)** Statistics showing the averaged change of basal [Ca^2+^]_i_ by cariporide and acetate. ***P < 0.0001.

To determine whether the cariporide effects on [Ca^2+^]_i_ are associated with cariporide-induced intracellular acidosis as shown in Fig. 6, we compared the effect of sodium acetate, a membrane-permeable weak acid, to that of cariporide in the same cells (Fig. 8). Figure 8A shows the effects of 1 µM cariporide and 20 mM sodium acetate on the pHi transients recorded from a representative experiment (an average of 15 cells). The result indicates that both cariporide and acetate produced intracellular acidosis, with acetate evoking a larger and faster acidification than cariporide.

Figure 8B compares the effect of cariporide and acetate on basal [Ca^2+^]_i_ and 20 K^+^-induced Ca^2+^ rise (an average of 15 cells). In contrast to the opposite effects of cariporide on increasing basal [Ca^2+^]_i_ but decreasing 20 K^+^-induced Ca^2+^ rise (see also Fig. 7), acetate lowered both basal [Ca^2+^]_i_ (marked by arrowhead) and 20 K^+^-induced Ca^2+^ rise. Figure 8C superimposes the Ca^2+^ transients to show the inhibition of peak amplitude and the lack of effect on the fast decay by cariporide (left panel) and acetate (right panel). Inset shows the opposite response of basal [Ca^2+^]_i_ to the application (marked by arrow) of cariporide and acetate.

Figure 8 *D1* shows the histogram for the distribution of cariporide- and acetate-induced percent change in Ca^2+^ transients (*n* = 154 cells from 8 experiments). In contrast to the almost all suppressive effect of cariporide (black bars), which reduced (by more than 10% of) the peak Ca^2+^ transient in 82% (126 out of 154) cells, acetate (grey bars) may suppress or enhance it in different SCN neurones, with 49% (75/154) being suppressed and 21% (33/154) enhanced by acetate. On average, cariporide and acetate reduced the peak Ca^2+^ transients by 19 ± 1% (*n* = 154 cells) and 8 ± 2% (*n* = 154 cells; t_(153)_ = 16.1, *P* < 0.0001, paired *t*-test) of control, respectively (Fig. 8D2). Together the results indicate that the smaller average inhibition by acetate is apparently due to its enhancing effect as observed in some cells.

Figure 8 *E1* shows the histogram for the distribution of cariporide- and acetate-induced change in basal [Ca^2+^]_i_, indicating mostly small, increasing effect of cariporide (black bars) and mostly larger, suppressive effect of acetate (grey bars) on basal [Ca^2+^]_i_. On average, 1 µM cariporide increased basal [Ca^2+^]_i_ by 0.0033 ± 0.0006 (*n* = 154 cells), in contrast to a decrease of 0.019 ± 0.001 (*n* = 154 cells; t_(153)_ = 6.05, *P* < 0.0001, paired *t*-test) by 20 mM acetate (Fig. 8E2).

### Nimodipine effects on Ca^2+^ responses to acetate and cariporide

**Figure 9:**
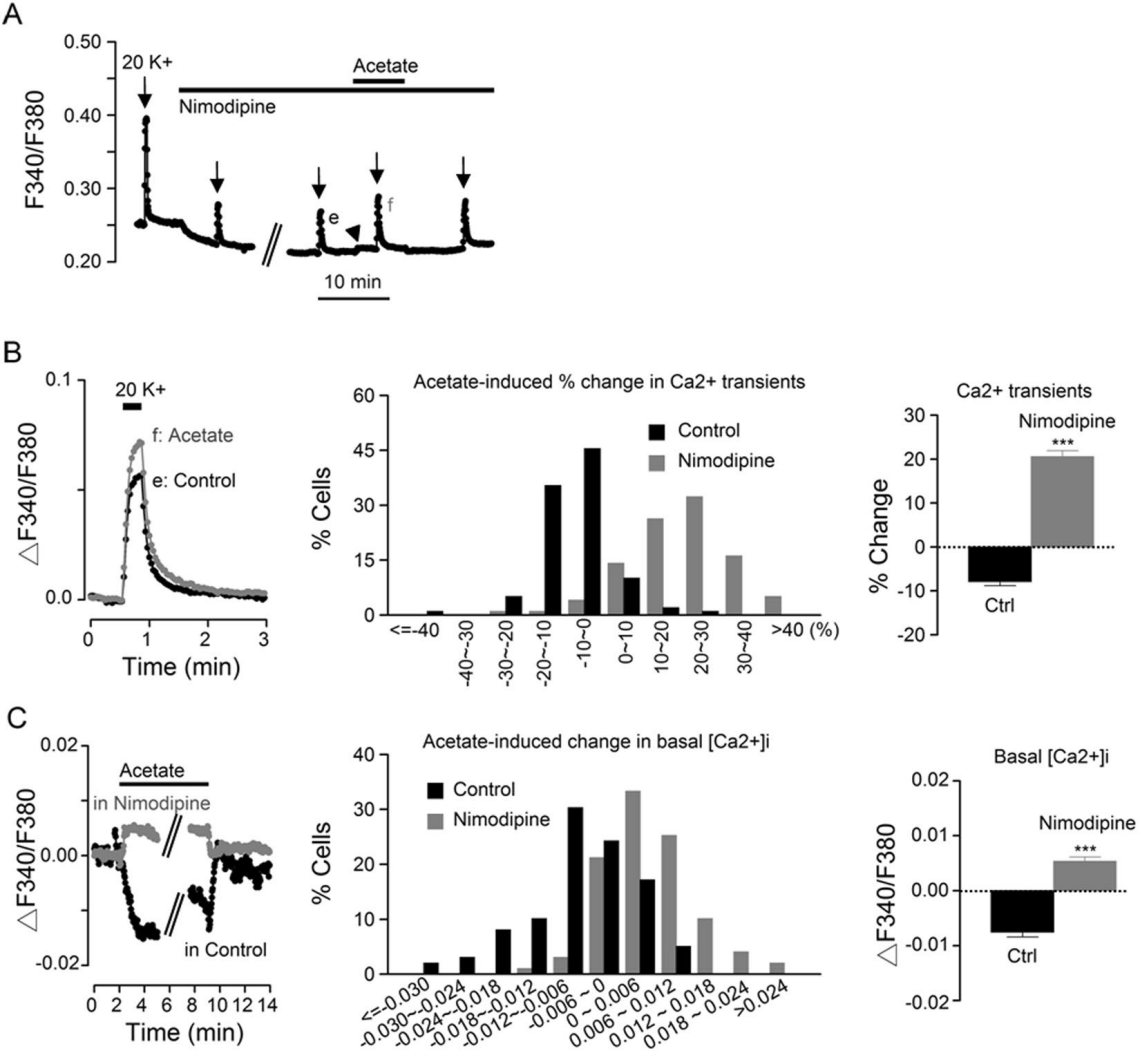
The effect of nimodipine on Ca^2+^ responses to sodium acetate. **(A)** A representative experiment showing the effect of 20 mM sodium acetate on [Ca^2+^]_i_ in the presence of 2 µM nimodipine (same experiment as in Fig. 8B). Note the increase of basal [Ca^2+^]_i_ by acetate (marked by arrowhead). **(B)** Superimposition of the Ca^2+^ transients showing the enhancing effect of acetate in the presence of nimodipine (*left*), as opposed to the suppressive effect of acetate in the absence of nimodipine (see Fig. 8C; right). *Middle*: Histogram showing the distribution of acetate-induced percentage changes in Ca^2+^ transients in control (black bars) and in the presence of nimodipine (grey bars) (*n* = 99 cells from 5 experiments). *Right*: Statistics showing that nimodipine converted acetate inhibition to enhancement of the peak amplitude of 20 K^+^-induced Ca^2+^ rise. **(C)** Superimposition of Ca^2+^ traces to indicate the increasing effect on basal [Ca^2+^]_i_ of acetate in the presence of nimodipine (grey filled circles), as opposed to the decreasing effect in control (black filled circles; from Fig. 8B, the Ca^2+^ response marked by arrowhead) (*right*). *Middle*: Histogram showing the distribution of acetate-induced changes in basal [Ca^2+^]_i_ transients in control (black bars) and in the presence of nimodipine (grey bars). *Right*: Statistics showing that nimodipine converted acetate-induced decrease to increase of basal [Ca^2+^]_i_. ***P < 0.0001.

As Ca^2+^ entering the nimodipine-sensitive L-type CaV1.2 channels is a major contributor to basal Ca^2+^ influx (determined with Ca^2+^-free solution) and to 20 K^+^-induced Ca^2+^ rise^29^, we reasoned that they might be involved in the suppressive effects of acetate on both basal [Ca^2+^]_i_ and 20 K^+^-induced Ca^2+^ rise. Indeed, 2 µM nimodipine converted the suppressive effects of acetate (Fig. 8B) to become stimulatory on both basal [Ca^2+^]_i_ (marked by arrowhead) and 20 K^+^-induced Ca^2+^ rise as indicated in Fig. 9A (same experiment as shown in Fig. 8B). Figure 9B, left panel, superimposes the Ca^2+^ transients to indicate an enhancing effect of acetate in the presence of nimodipine, as opposed to a suppressive effect in its absence (cf. Figure 8C, right panel). Middle panel shows the histogram for the distribution of acetate-induced percent change in Ca^2+^ transients in control (black bars) and in nimodipine (grey bars), indicating a nimodipine-induced shift from mostly suppressive to stimulatory action of acetate (*n* = 99 cells from 5 experiments). On average, acetate reduced the peak Ca^2+^ transients by 8 ± 1% (*n* = 99 cells) in control but increased it by 20 ± 1% (*n* = 99 cells; t_(98)_ = 17.98, *P* < 0.0001, paired *t*-test) in the presence of nimodipine (right panel).

Similarly, superimposition of basal [Ca^2+^]_i_ also indicates an increasing effect of acetate in the presence of nimodipine (grey circles) in contrast to a decreasing effect in its absence (dark circles) (Fig. 9C, left panel). Middle panel shows the histogram for the distribution of acetate-induced change in basal [Ca^2+^]_i_ in control (black bars) and in nimodipine (grey bars), again indicating a nimodipine-induced shift from mostly decreasing to increasing effect of acetate (*n* = 99 cells). On average, acetate lowered the basal [Ca^2+^]_i_ by 0.0075 ± 0.0010 (*n* = 99 cells) in control but increased it by 0.0054 ± 0.0008 (*n* = 99 cells; t_(98)_ = 10.13, *P* < 0.0001, paired *t*-test) in the presence of nimodipine (right panel). Together with Fig. 8, the results indicate that acetate inhibits nimodipine-sensitive, but enhances nimodipine-insensitive, basal [Ca^2+^]_i_ and 20 K^+^-induced Ca^2+^ rise.

**Figure 10:**
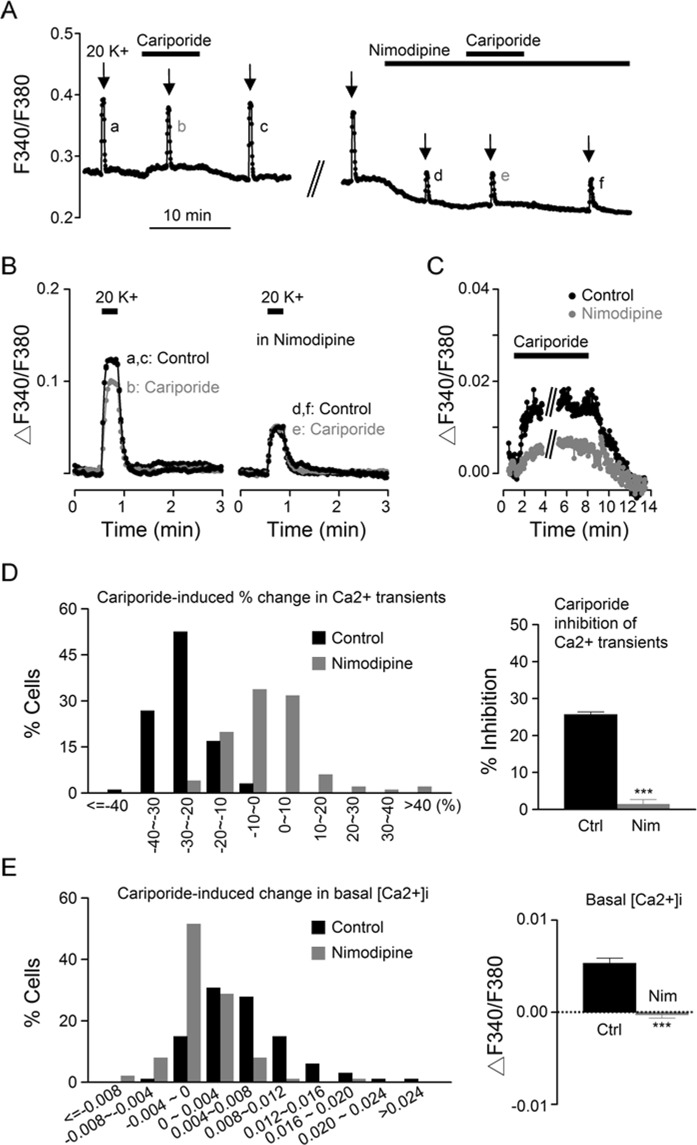
The effect of nimodipine on Ca^2+^ responses to cariporide. **(A)** A representative experiment showing the effect of 1 µM cariporide on [Ca^2+^]_i_ in the absence and then the presence of 2 µM nimodipine. (**B**) Superimposition of the Ca^2+^ transients to show that the suppressive effect of cariporide (*left*) was abolished in the presence of nimodipine (*right*). (**C**) Superimposition of Ca^2+^ traces to indicate that cariporide-induced increase in basal [Ca^2+^]_i_ (black filled circles) became smaller in the presence of nimodipine (grey filled circles). **(D)** Histogram showing the distribution of cariporide-induced percentage changes in Ca^2+^ transients in control (black bars) and in the presence of nimodipine (grey bars) (*n* = 101 cells from 5 experiments) (*left*). *Right*: Statistics showing that cariporide-induced inhibition of the peak amplitude of 20 K^+^-induced Ca^2+^ rise was abolished by nimodipine. **(E)** Histogram showing the distribution of cariporide-induced changes in basal [Ca^2+^]_i_ in control (black bars) and in the presence of nimodipine (grey bars) (*left*). *Right*: Statistics showing that cariporide-induced increase of basal [Ca^2+^]_i_ was also abolished by nimodipine. ***P < 0.0001.

To determine whether the nimodipine-sensitive L-type Ca^2+^ channels may also be involved in mediating cariporide effects, the experiments were performed with cariporide applied in the absence and then the presence of 2 µM nimodipine as indicated in Fig. 10A. Superimposition of Ca^2+^ transients in the absence (left panel) and presence (right panel) of nimodipine indicate that nimodipine virtually eliminated the cariporide effect on the Ca^2+^ transient (Fig. 10B), whereas superimposition of basal [Ca^2+^]_i_, nimodipine also markedly, but incompletely, reduced the cariporide effect on basal [Ca^2+^]_i_ for this particular experiment (Fig. 10C).

Figure 10D, left panel, shows the histogram for the distribution of cariporide-induced percent change in Ca^2+^ transients in control (black bars) and in nimodipine (grey bars), indicating a nimodipine-induced shift from mostly inhibition to a distribution centered around zero inhibition for cariporide (a total of 101 cells from 5 experiments). On average, cariporide reduced the peak Ca^2+^ transients by 26 ± 1% (*n* = 101 cells) in control and 1 ± 1% (*n* = 101 cells; t_(100)_ = 15.98, *P* < 0.0001, paired *t*-test) in the presence of nimodipine (right panel). Figure 10E, left panel, shows the histogram for the distribution of cariporide-induced change in basal [Ca^2+^]_i_ in control (black bars) and in nimodipine (grey bars), again indicating a nimodipine-induced shift from positive-going changes to a distribution centered around no change in basal [Ca^2+^]_i_. On average, cariporide changed the basal [Ca^2+^]_i_ by 0.0053 ± 0.0006 (*n* = 101 cells) in control and −0.0003 ± 0.0004 (*n* = 101 cells; t_(100)_ = 11.27, *P* < 0.0001, paired *t*-test) in the presence of nimodipine (right panel). The abolition of cariporide effects by nimodipine suggests that the effects of cariporide on basal [Ca^2+^]_i_ and 20 K^+^-induced Ca^2+^ rise were mediated via the nimodipine-sensitive L-type Ca^2+^ channels.

### NHE1 localisation in the SCN

**Figure 11:**
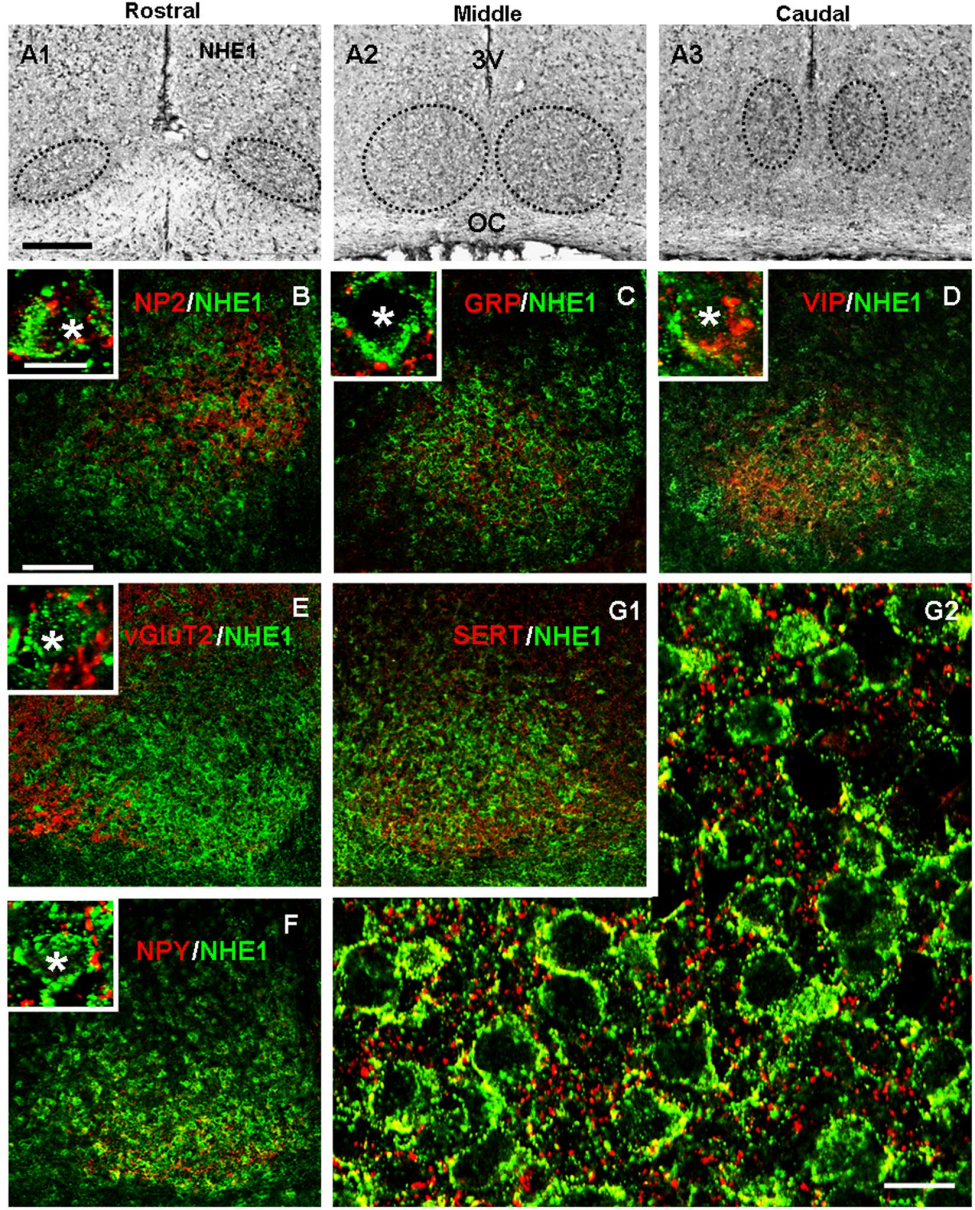
NHE1 distribution (**A**) and colocalisation with markers for specific cell types (**B**–**D**) and major inputs (**E**, **F**, **G**). **(A)** NHE1 immunoreactivity is distributed throughout the rostrocaudal axis of the SCN (encircled by the dotted lines). Scale bar: 200 µm. OC: optic chiasm. 3 V: third ventricle. **(B*–*G1)** Low magnification images showing the double staining pattern of NHE1 with neuropeptides NP2 (**B**), GRP (**C**), and VIP (**D**) as well as markers for afferent inputs vGluT2 (**E**), NPY (**F**), and SERT (**G1**). Scale bar: 100 µm. Insets: High magnification images showing individual cells with double staining. Scale bar: 10 µm. Asterisks mark Hoechst-stained nuclei. **(G2**) High magnification image showing high degree of colocalisation (yellow) between NHE1 (green) and SERT (red). Scale bar: 10 µm.

Immunohistochemistry with the NHE1-specific antibody was used to study the distribution pattern of the NHE1 isoform (Fig. 11A). The result shows the presence of NHE1 immunoreactivity throughout the rostrocaudal axis of the SCN (Fig. 11A1–A3) and in the medial SCN the NHE1 immunoreactivity is present in the whole SCN (Fig. 11A2). The high magnification image revealed punctate or aggregate localisation of NHE1 (green) around the soma (see Fig. 11B–F*, insets* and Fig. 11G2). To determine the localisation of NHE1 in the specific type of cells, double staining immunofluorescence for NHE1 and the three major neuropeptides AVP-partner NP2, GRP, and VIP were performed in the mid-SCN sections (Fig. 11B–D). The results show a lack or very low degree of colocalisation (yellow) with NP2 (Fig. 11B), GRP (Fig. 11C), or VIP (Fig. 11D).

To determine the possible presence of NHE1 in afferent inputs to the SCN, antibodies for the vesicular glutamate transporter type 2 (vGluT2), neuropeptide Y (NPY), or serotonin transporter (SERT) were used to perform double staining with NHE1 (Fig. 11E,F,G1). The results indicate a lack of colocalisation with vGluT2 (Fig. 11E), albeit with immunostained puncta closely opposing the NHE1-immunoreactive soma (inset), and a low degree of colocalisation (yellow) with NPY (Fig. 11F). In contrast, there was a high level of colocalisation with SERT (Fig. 11G2), with punctate double staining near the cell membrane and aggregated in the neuropil (Fig. 11G2). Note the SERT-immunoreactive broken fibres (red) coursing between the cells and contacting the cell to reveal punctate double stain (yellow) with NHE1 (green).

**Figure 12:**
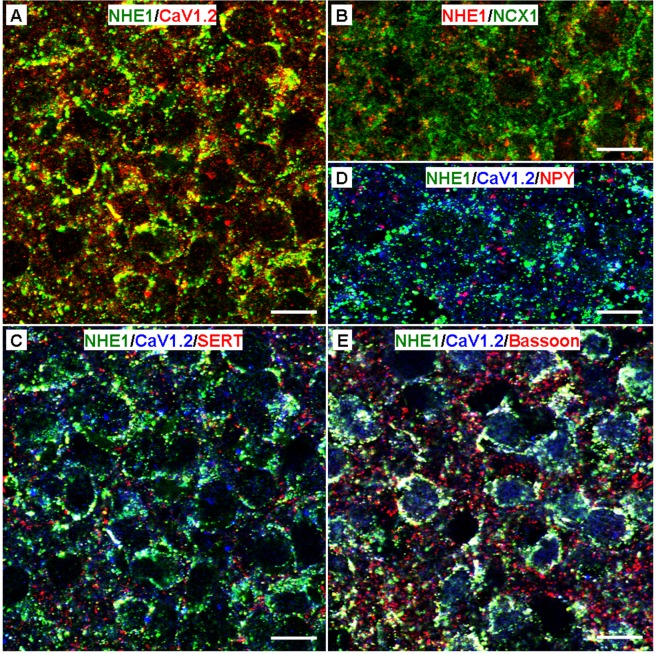
NHE1 colocalisation with CaV1.2 (**A**) and NCX1 (**B**), as well as colocalisation of NHE1/CaV1.2 with SERT (**C**), NPY (**D**), and Bassoon (**E**). (**A**, **B**) High magnification images showing high levels of NHE1 colocalisation with CaV1.2 (**A**), but not NCX1 (**B**). (**C**–**E**) High magnification images showing moderate to high levels of NHE1/CaV1.2 colocalisation with SERT (**C**) and Bassoon (**E**), but not NPY (**D**). Scale bar: 10 µm.

We also determined the colocalisation of NHE1 with CaV1.2 and NCX1, two major plasmalemmal proteins involved in shaping 20 mM K^+^-evoked somatic Ca^2+^ rise and fast Ca^2+^ decay^28–30^. Our immunofluorescent double staining showed a high degree of colocalisation (yellow) between NHE1 (green) and CaV1.2 (red) (Fig. 12A), but not between NHE1 (red) and NCX1 (green) (Fig. 12B).

To further assess the possible involvement of NHE1 and CaV1.2 in the regulation of neurotransmission, we performed triple immunostaining to determine the colocalisation of NHE1, CaV1.2, and SERT (Fig. 12C) or NPY (Fig. 12D). The results indicate a moderate to high level of triple staining (white) for NHE1 (green), CaV1.2 (blue), and SERT (red), mostly around the cell membrane (Fig. 12C), but a very low level of triple staining (white) for NHE1 (green), CaV1.2 (blue), and NPY (red) (Fig. 12D). Note that Fig. 12C and Fig. 12A are from the same area of the same section, with different colour for CaV1.2 stain. Figure 12E shows the intense triple staining (white) for NHE1 (green), CaV1.2 (blue), and the presynaptic cytomatrix protein Bassoon (red), a marker for presynaptic active zone (for review see ref.^31^) and varicosity^32^. Together the results indicate high levels of colocalisation between NHE1 and CaV1.2 both in the soma and in the presynaptic structures including at least the serotonergic input.

## Discussion

This study demonstrates a role of NHE1 in the regulation of pHe, pHi, and [Ca^2+^]_i_ in the SCN clock. We show that NHE1 actively extrudes H^+^ to contribute to standing extracellular acidification in the SCN. The constitutive activation of NHE1 maintains a more alkaline pHi in the soma and regulates somatic [Ca^2+^]_i_ by functional coupling with the nimodipine-sensitive L-type Ca^2+^ channels, in accordance with punctate colocalisation of NHE1 with CaV1.2 around the cell membrane. NHE1 is also present in presynaptic structures, particularly associated with serotonergic inputs. The triple staining of NHE1, CaV1.2 and SERT or Bassoon suggests that NHE1, along with CaV1.2, may also regulate presynaptic Ca^2+^ levels and, perhaps at least serotonergic, neurotransmission in the SCN.

We show a standing pHe gradient between the SCN and the superfusion solution, adding the SCN to the list of neural tissues with more acidic pHe (see ref.^16^). The maximum acidification occurs at the center of 300-μm hypothalamic slices and amounts to ~0.3 pH units in a depth of 150 µm from the surface of the SCN superfused with 10 mM HEPES-buffered solution at pH 7.4. The more acidic pHe measured in the SCN compared with the surrounding extra-SCN areas is most likely due to the higher density packing of SCN neurones along with higher level of metabolic activity than extra-SCN areas^8^. As such more protons are produced in conjunction with more restricted proton diffusion due to reduced extracellular space in the SCN.

The NHE blocker amiloride dose-dependently increases the pHe with an IC_50_ of 30 µM. While the SCN expresses mRNAs for the plasmalemmal-type NHE1, NHE4, and NHE5 isoforms, the NHE1-specific antagonist cariporide dose-dependently increases the pHe with an IC_50_ of 0.09 µM, a value consistent with the published data (IC_50_ ~0.03–3.4 µM)^23^. Together the result reveals that NHE1 actively extrudes H^+^ to contribute to the standing extracellular acidification in the SCN. As immunostaining localizes NHE1 to the cell membrane and the presynaptic structures, particularly associated with the serotonergic input, the result suggests that the standing extracellular acidification in the SCN is partly mediated by H^+^ extruded via NHE1 from both pre- and post-synaptic regions.

Consistent with the result of cariporide-sensitive (i.e. NHE1-mediated) H^+^ extrusion, ratiometric intracellular H^+^ imaging also shows cariporide-induced intracellular acidifications in the SCN cells. Together, our results indicate that constitutive activation of NHE1 maintains a more alkaline pHi and contributes to extracellular acidification in the SCN. Since NHE1 is allosterically activated by intracellular H^+1^ ^27^, the constitutive activation of NHE1 is most likely due to high rate of proton production associated with high metabolic activity of the SCN, as determined with ^14^C-2-deoxyglucose method, compared to other regions of brain^8,33,34^.

The ability of NHE1 to maintain a more alkaline pHi plays a role in regulating [Ca^2+^]_i_ in the SCN cells. We show that cariporide blockade of NHE1 increases basal [Ca^2+^]_i_ but decreases depolarisation (20 mM K^+^)-induced Ca^2+^ rise. The cariporide effect on [Ca^2+^]_i_ appears to be closely associated with the Ca^2+^ entering the L-type Ca^2+^ channels, because 2 µM nimodipine abolishes the cariporide effect on both basal [Ca^2+^]_i_ and depolarisation-induced Ca^2+^ rise. In contrast, the weak acid acetate, which also induces intracellular acidosis, decreases both basal [Ca^2+^]_i_ and depolarisation-induced Ca^2+^ rise. Nimodipine converts acetate inhibition to enhancement of basal [Ca^2+^]_i_ and 20 K^+^-induced Ca^2+^ rise, suggesting that intracellular acidosis evoked by acetate has opposite effects on nimodipine-sensitive and -insensitive [Ca^2+^]_i_, being suppressive for the former and stimulatory for the latter.

It is not possible at this moment to provide a detailed account for the results, because intracellular Ca^2+^ homeostasis relies on the functional interaction of various Ca^2+^ handling systems involved in mediating Ca^2+^ entry, extrusion, and buffering, all of them affected by intracellular acidosis. For example, intracellular H^+^ inhibits voltage-dependent Ca^2+^ influx, internal Ca^2+^ release from the ryanodine receptor, forward NCX activity, and mitochondrial uptake via the Ca^2+^ uniporter (see ref.^35^ and references therein). Nevertheless, our recent findings suggest the possible involvement of L-type Ca^2+^ channels and mitochondrial uptake of Ca^2+^ entering nimodipine-insensitive Ca^2+^ channels. First, we show that Ca^2+^ entering L-type Ca^2+^ channels is a major contributor to basal Ca^2+^ influx (determined with Ca^2+^-free solution) and to 20 K^+^-induced Ca^2+^ rise^29^. Second, mitochondria appear to regulate [Ca^2+^]_i_ by buffering Ca^2+^ entering nimodipine-insensitive Ca^2+^ channels, including N-, P/Q-, and most likely also T-type Ca^2+^ channels^28,29^. Together, a simple explanation suggests that acetate-induced acidosis inhibits L-type Ca^2+^ channels to account for the inhibition of nimodipine-sensitive basal [Ca^2+^]_i_ and depolarisation-induced Ca^2+^ rise, and also inhibits mitochondrial Ca^2+^ uptake to account for the enhancement of nimodipine-insensitive basal [Ca^2+^]_i_ and depolarisation-induced Ca^2+^ rise^29^.

Unlike the parallel inhibition by acetate of nimodipine-sensitive basal [Ca^2+^]_i_ and Ca^2+^ transients, cariporide inhibits nimodipine-sensitive Ca^2+^ rise and yet increases nimodipine-sensitive basal [Ca^2+^]_i_. It is beyond the scope of this study to elucidate the underlying mechanisms for the opposite effects of cariporide on nimodipine-sensitive basal [Ca^2+^]_i_ and 20 K^+^-induced Ca^2+^ rise. Nonetheless, the mechanism most likely lies in the discrete localisation of NHE1 and its regulation of local pH around specific Ca^2+^ handling proteins, as opposed to the more homogenous change in the pHi induced by acetate. The combined results of Ca^2+^ imaging and immunostaining suggest that NHE1 could regulate local pH around the CaV1.2 channel to influence its activity and thus associated Ca^2+^ signaling. Similarly specific targeting of NHE1 to distinct regions of the cell membrane has been demonstrated in both atrial and ventricular muscle cells, with NHE1 predominantly localized to the intercalated disk region close to the gap junction protein connexion 43^36^. Since gap junction conductance is sensitive to small change in the physiological pHi^37,38^, NHE1 regulation of local pH around the gap junction could regulate intercellular communication.

The ability of NHE1 to regulate both intra- and extra-cellular pH in the SCN suggests that NHE1 may play a multitude of roles in the regulation of circadian clock. Here we demonstrate a role of NHE1 in regulating intracellular Ca^2+^ handling associated with nimodipine-sensitive Ca^2+^ channels. In view of an important role of L-type Ca^2+^ channels in regulating PER2 oscillation^39^ and light/glutamate-induced phase advance^40,41^, it would be interesting to see whether NHE1 may play a role in the regulation of clock gene expression and light-induced phase shifts.

The same NHE1 regulation of pHi and [Ca^2+^]_i_ in the soma may also be at work in the presynaptic terminals to regulate neurotransmission, as suggested by the colocalisation of NHE1/CaV1.2 with Bassoon and, more specifically, the serotonergic input. The results may be taken to suggest the involvement of NHE1 along with CaV1.2 in regulating presynaptic Ca^2+^ levels and, perhaps at least serotonergic, neurotransmission. As serotonergic signaling via presynaptic 5HT-1B receptor is known to inhibit light (glutamate)-induced phase shifts (for review, see ref.^42^) and, more profoundly, GABAergic neurotransmission in the SCN (see ref.^43^ and references therein), NHE1 along with CaV1.2 could potentially regulate glutamatergic and GABAergic neurotransmission. Further work is warranted to determine whether and how NHE1 and CaV1.2 play a role in the regulation of serotonergic, glutamatergic, and GABAergic neurotransmission.

On the other hand, although it is not known how NHE1-mediated extracellular acidification may play a role in SCN physiology, our previous study on acid-sensing ion channels (ASIC) indicates that the SCN neurones are sensitive to regulation by extracellular pH^18^. In particular, the SCN neurones express ASIC channels that contain high pH sensitivity of ASIC3 and ASIC1a subunits, with ~95% of them excited by neutral pH (pH 7.0). Furthermore, membrane conductances involved in membrane excitability and neurotransmission such as NMDA receptors, GABA_A_ receptors, and voltage-gated calcium channels are all sensitive to extracellular protons^4,16^ and play a role in the regulation of circadian clock (see ref.^19^). Indeed, our unpublished observation show that lowering extracellular pH from 7.4 to 7.1, mimicking the more acidic pHe in the center of SCN slices, altered neuronal excitability and [Ca^2+^]_i_ in the SCN neurones in reduced slice preparations. Further work is needed to obtain more detailed knowledge of pH regulation in the SCN in order to better understand whether and how the more acidic pHe may play a role in the regulation of the circadian clock of the SCN.

In conclusion, the constitutively active NHE1 extrudes H^+^ to maintain a more alkaline pHi and a more acidic pHe in the SCN. NHE1, by functional coupling to L-type CaV1.2 channels, helps regulate somatic and, perhaps, presynaptic Ca^2+^ levels to play a role in the regulation of circadian clock of the SCN.

## Methods

### Hypothalamic brain slices and reduced SCN preparations

All experiments were carried out according to procedures approved by the Institutional Animal Care and Use Committee of Chang Gung University. Sprague-Dawley rats (18–24 days old) were kept in a temperature-controlled room under a 12:12 light:dark cycle (light on 0700–1900 hr). Lights-on was designated Zeitgeber time (ZT) 0. For daytime (ZT 4–11) and nighttime (ZT 13–20) recordings, the animal was killed at ZT 2 and ZT 10, respectively. Hypothalamic brain slices and reduced SCN preparations were made as described previously^28,29^. An animal of either sex was carefully restrained by hand to reduce stress and killed by decapitation using a small rodent guillotine without anaesthesia, and the brain was put in an ice-cold artificial cerebrospinal fluid (ACSF) prebubbled with 95% O_2_-5% CO_2_. The ACSF contained (in mM): 125 NaCl, 3.5 KCl, 2 CaCl_2_, 1.5 MgCl_2_, 26 NaHCO_3_, 1.2 NaH_2_PO_4_, 10 glucose. A coronal slice (200–300 µm) containing the SCN and the optic chiasm was cut with a DSK microslicer DTK-1000 (Ted Pella, Redding, CA, USA), and was then incubated at room temperature (22–25 °C) in the incubation solution, which contained (in mM): 140 NaCl, 3.5 KCl, 2 CaCl_2_, 1.5 MgCl_2_, 10 glucose, 10 HEPES, pH 7.4, bubbled with 100% O_2_.

For fluorescent Ca^2+^ and H^+^ imaging, a reduced SCN preparation was obtained by excising a small piece of tissue (circa one-ninth the size of SCN) from the medial SCN using a fine needle (Cat no. 26002-10, Fine Science Tools, Foster City, CA, USA), followed by further trimming down to 4–10 smaller pieces with a short strip of razor blade. The reduced preparation (containing tens of cells, see Fig. 6) was then transferred to a coverslip precoated with poly-D-lysine (Sigma-Aldrich, St Louis, MO, USA) in a recording chamber for recording. The SCN cells of the reduced preparation could be identified visually with an inverted microscope (Olympus IX70 and IX71, Japan). The preparation thus obtained allows rapid application of drugs^18^ and has been used for Na^+^ and Ca^2+^ fluorescent imaging^12,28^ and to demonstrate diurnal rhythms in both spontaneous firing and Na/K pump activity^10^.

### Extracellular pH measurements in hypothalamic slices

Extracellular pH in the SCN was measured with double-barreled pH-selective microelectrodes based on established methods^44,45^. The microelectrodes were pulled from double-barreled borosilicate glass capillaries with filament (2BF100-50-10, Sutter, Novato, CA, USA) with a vertical pipette puller (PE-21, Narishige, Japan). The tips were broken to a diameter of ~10 µm. The pH-selective barrel was selectively silanized with N,N-dimethyltrimethylsilylamine (Fluka 41716, Sigma-Aldrich, St Louis, MO, USA) according to a modified method^46^. The reference and pH-selective barrels were backfilled with a solution containing (in mM): 100 NaCl, 20 HEPES, 10 NaOH, pH 7.4. Positive pressure was applied to the back of pH-selective barrel to ensure a good backfilling. A column of hydrogen ionophore I-cocktail A (Fluka 95291; Sigma-Aldrich, St Louis, MO, USA) was then drawn into the tip of pH-selective barrel with or without suction. The electrode was calibrated before each experiment in a series of standard solutions (see Fig. 1), having an averaged slope response of −55.7 ± 0.7 mV per unit pH change (*n* = 71 electrodes). The resistance of the pH-selective barrel was 5–10 GΩ, whereas the reference barrel had a resistance between 20 and 50 MΩ. All recordings were made with a Duo 773 Electrometer (World Precision Instruments, Sarasota, FL, USA) at room temperature (22–25 °C), with the signal low-pass filtered at 1 kHz and digitized online at 2 kHz with a PowerLab 4/30 (ADInstruments, Dunedin, New Zealand).

Earlier experiments used HCO_3_^−^ buffered solution containing (in mM): 124 NaCl, 3 KCl, 26 NaHCO_3_, 1.0 NaH_2_PO_4_, 2.5 CaCl_2_, 1.5 MgCl_2_, and 10 glucose, equilibrated with 95% O_2_ and 5% CO_2_ (pH = 7.40). In some experiments the NaHCO_3_ was increased to 35 mM and NaCl reduced correspondingly to provide a bath pH of 7.55 (Fig. 2B). All the rest of experiments used HEPES-buffered solution that contains (in mM): 140 NaCl, 3.5 KCl, 2 CaCl_2_, 1.5 MgCl_2_, 10 HEPES, 10 glucose, pH 7.4.

### Ca^2+^ and H^+^ imaging in reduced SCN preparations

Ratiometric fluorescence imaging was carried out as described previously^28,29^. Fluorescent Ca^2+^ and H^+^ imaging was performed, respectively, by pre-loading the SCN cells with the Ca^2+^-sensitive fluorescent indicator Fura2-acetoxymethyl ester (Fura2-AM)^47^ and the proton-sensitive fluorescent indicator 2′,7′-bis-(2-carboxyethyl)-5-(and-6)-carboxyfluorescein acetoxymethyl ester (BCECF-AM)^48^. The reduced SCN preparation was incubated in 10 µM Fura2-AM or 2 µM BCECF-AM in 50 µl of bath solution in the dark for 60 min at 37 °C. Incubation was terminated by washing with 6 ml of bath solution and at least 60 min was allowed for de-esterification of the dye. All imaging experiments were performed at room temperature (22–25 °C). For the experiments, the reduced SCN preparation was gently pressed on the edge against the coverslip to allow adherence of the tissue to the surface. Fluorescence signals were imaged using a charge-coupled device camera attached to an inverted microscope (Olympus IX71, Japan) and recorded with Xcellence imaging software integrated with the CellIR MT20 illumination system (Olympus Biosystems, Planegg, Germany). The system used a 150-W xenon arc burner as the light source to illuminate the loaded cells. The excitation wavelengths were 340 (±12) and 380 (±14) nm (for Ca^2+^) or 440 (±24) and 490 (±20) nm (for H^+^) and emitted fluorescence was collected at 510 nm. Pairs of 340/380 nm and 440/490 nm images were, respectively, sampled at 0.5 and 0.1 Hz. Ca^2+^ or H^+^ levels in regions of interest (ROI) over the soma were spatially averaged and presented by fluorescence ratios (F340/F380 for Ca^2+^ and F440/F490 for H^+^) after background subtraction.

### Drugs

Stock solutions of nimodipine (20 mM in DMSO), amiloride (500 mM in DMSO), and cariporide (10 mM in DMSO) were stored at −20 °C, and were diluted at least 1000 times to reach desired final concentrations. Nimodipine and cariporide were purchased from Tocris Cookson (Ellisville, MO, USA), and amiloride from Sigma-Aldrich (St Louis, MO, USA). Na^+^-free solutions were prepared with total replacement of extracellular Na^+^ with N-methyl-D-glucamine (NMDG^+^), and 20 mM K^+^ solutions were prepared with equal molar substitution of K^+^ for Na^+^. All solutions were adjusted to pH 7.4 before use.

### Histology, immunohistochemistry, and immunofluorescence

Immunohistochemistry and immunofluorescence staining were performed as described previously^28,29^_._ Sprague-Dawley rats (23–25 days old) were deeply anesthetized with Zoletil (40 mg/kg, i.p.; Virbac Laboratories, Carros, France) and fixed by transcardial perfusion with PBS and then with 4% paraformaldehyde (500 ml/animal). Brains were removed and post-fixed overnight (more than 16 hr) in 4% paraformaldehyde, followed by dehydration with 30% sucrose in PBS for another 24 hr. Twenty-micrometer-thick coronal sections through the hypothalamus region containing the SCN were cut on a cryostat (−20 °C), collected in antifreeze solution, and stored in −20 °C freezer until further processing.

For Nissl staining, sections (20 µm) were washed for 20–30 min in PBS and then stained with 1% Nissl (cresyl violet acetate) (C5042, Sigma, St. Louis, MO, USA). After gradient ethanol hydration, sections were coverslipped with DPX (101979, Merck Millipore, Billerica, MA, USA) and photographed using an inverted microscope (Olympus IX71, Japan).

For immunohistochemical staining, sections (20 µm) were treated with 0.3% H_2_O_2_ for 15 min to quench endogenous peroxidase, and then incubated overnight at 4 °C in PBS containing 2% serum, 0.3% Triton X-100, and primary antibodies NHE1 (rabbit anti-NHE1; 1:200; ab67314, RRID:AB_1141782; Abcam, Cambridge, MA, USA). Sections were then treated with respective biotinylated secondary antibodies for 1 h at room temperature (22–25 °C). After rinsed in PBS, sections were then incubated in avidin-biotin complex (ABC Elite Kit, Vector Labs, Burlingame, CA, USA) for 1 h according to the manufacturer’s instructions. After two 10-min washes in 0.1 M sodium acetate, sections were stained with diaminobenzidine. Sections were photographed and analyzed with an inverted microscope (Olympus IX71, Japan) integrated with the MT20 illumination system (Olympus Biosystems, Planegg, Germany).

For immunofluorescence staining, sections (20 µm) were washed for 20–30 min in PBS and then incubated overnight at 4 °C in PBS containing 2% serum, 0.3% Triton X-100, and primary antibodies against NHE1 (rabbit anti-NHE1; 1:100; ab67314, RRID:AB_1141782; Abcam, Cambridge, MA, USA), neurophysin II (NP2) (goat anti-NP2; 1:500; sc-27093, RRID:AB_2061964; Santa Cruz, CA, USA), vasoactive intestinal peptide (VIP) (guinea pig anti-VIP; 1:500; T-5030, RRID:AB_518690; Peninsula Laboratories, San Carlos, CA, USA), gastrin-releasing peptide (GRP) (goat anti-GRP; 1:100; sc-7788, RRID:AB_2232721; Santa Cruz, CA, USA), vesicular glutamate transporter type 2 (vGluT2) (guinea pig anti-vGluT2; 1:300; AB2251, RRID:AB_1587626; Millipore, Temecula, CA, USA), serotonin transporter (SERT) (mouse anti-SERT; 1:200; MAB1564, RRID:AB_94220; Millipore, Temecula, CA, USA), neuropeptide Y (NPY) (goat anti-NPY; 1:300; NBP1-46535, RRID:AB_10009813; Novus, Littleton, CO, USA), CaV1.2 (guinea pig anti-CaV1.2; 1:100; AGP-001; RRID:AB_11219156; Alomone Labs, Jerusalem, Israel), NCX1 (mouse anti-NCX1, against epitope between amino acid 371 and 525 on intracellular side of plasma membrane; 1:100; AB2869, RRID:AB_2191134; Abcam, MA, USA), and Bassoon (mouse anti-Bassoon; 1:200; ADI-VAM-PS003-D, RRID:AB_2038857; Enzo Life Sciences, Farmingdale, NY, USA). Sections were then treated with respective Alexa Fluor secondary antibodies 488, 568, or 633 (1:300 for Alexa Fluor 488 and 568, 1:200 for Alexa Fluor 633; Molecular Probes, Eugene, OR, USA) and Hoechst 33342 (B-2261; Sigma, St. Louis, MO, USA) for 1 hr at room temperature. After rinse in PBS, sections were coverslipped with ProLong Gold anti-fade reagent (P36930; Molecular Probes, Eugene, OR, USA) and photographed with Zeiss LSM 510 confocal microscope. Contrast and brightness were optimized using Adobe Photoshop (Adobe Systems, San Jose, CA, USA).

### RT-PCR analysis of NHE1–5 expression

RT-PCR was performed as described previously^28^. Total RNA of SCN was extracted using the Absolutely RNA Nanoprep kit (Stratagene, La Jolla, CA, USA) according to the manufacturer’s guide; total RNA of rat brain was purchased from BioChain Institute Inc (Newark, CA, USA). RNA samples were treated with DNaseI for 13–15 min at 25 °C to eliminate genomic DNA contamination. The resulting RNA was reverse-transcribed (RT) to cDNA using ReverTra Ace (TOYOBO, Osaka, Japan) with oligo(dT) primers in a total volume of 20 μl. One-tenth of RT products were used as templates (2 μl) to perform PCR reaction. RT reaction with omission of reverse transcriptase was used as templates for negative control PCR. Primers used for RT-PCR were as follows: NHE1 forward 5′-CACAGTTCCTGGACCACCTT-3′ and reverse 5′-GGATCTCCTCCTCCTTGTCC-3′, NHE2 forward 5′-TCTGCTTTGCACTGGTGTTC-3′ and reverse 5′-GATGCAAATGAGGGGACAGT-3′, NHE3 forward 5′-CCTTTCCGAATTGAAGTCC-3′ and reverse 5′-CGGCTGCTAGCTTTGGTATC-3′, NHE4 forward 5′-GGTGTGAGAGGAGCAGGAAG-3′ and reverse 5′-TAGCCCAGTCTCTGCCATCT-3′, and NHE5 forward 5′-CGTTAGGGGGCATTGTCTTA-3′ and NHE5 reverse 5′-TCAAAGACAGCCAACACAGC-3′. The thermal cycling condition of RT-PCR was 94 °C for 3 min, followed by 35 cycles of 94 °C for 30 s, 60 °C for 30 s, and 72 °C for 30 s, and then 72 °C for 7 min. PCR amplified products were electrophoresed in 1.5% agarose gels, stained with ethidium bromide, and photographed.

### Real-time PCR analysis of mRNA levels of *NHE1*, r*Per1*, and r*Per2*

Real-time PCR was performed as described previously^25^. Total mRNA of the SCN was prepared from freshly dissected tissue by extraction with Absolutely RNA Nanoprep kit (Stratagene, La Jolla, CA, USA) according to the manufacturer’s instructions. The purity of total RNA in each sample was measured using a microplate spectrophotometer reader (Multiskan GO; Thermo Fisher Scientific, USA). Samples were volume-adjusted with RNAase-free water and normalized for their RNA content. The resulting RNA was reverse-transcribed to cDNA with Superscript III reverse transcriptase (Invitrogen, Carlsbad, CA, USA) and oligo(dT)_12–18_ primers in a total volume of 30 µl.

The mRNA expression levels of *NHE1, rPer1*, and r*Per2* were measured by real-time PCR analysis with SYBR Green method. The target genes and *GAPDH* were amplified separately using the same group of cDNA template from each sample. Successful reverse transcription was confirmed for all samples by performing PCR amplification of the internal control *GAPDH*. Primer sequences for target genes were as follows: *GAPDH* forward 5′-GCATCTTCTTGTGCAGTGCC-3′ and reverse 5′-TACGGCCAAATCCGT TCACA-3′, *NHE1* forward 5′-CTGCAGTCGGACGTCTTCTT -3′ and reverse 5′- GTTCTCCGTGAACTGCCTCA -3′, *Per1* forward 5′-TGTGTGGACTGTGGTAGC-3′ and reverse 5′-TCTGAGAAGAGAGGGTCGT-3′, and *Per2* forward 5′-CCAGAGGCGAG- AGCTTC-3′ and reverse 5′-GATGGCGGTAGGCAGAC-3′. PCR amplification was carried out using 2 × Power SYBR Green PCR Master Mix (Applied Biosystems, Framingham, MA, USA) in the StepOne Real Time PCR System (48-well format) (Applied Biosystems, Framingham, MA, USA). The PCR reaction setup included 10 µl of 2 × Power SYBR Green PCR Master Mix, 0.6 µl of 10 µM forward primer, 0.6 µl of 10 µM reverse primer, and 2 µl (10 ng) of cDNA in a total reaction volume of 20 µl. Cycle threshold (C_T_) values were obtained from the exponential phase of PCR amplification. The 2^−ΔΔC^T method was used to calculate the mRNA levels normalized to the *GAPDH* (ΔC_T_ = target gene C_T_ − GAPDH C_T_)^49^.

### Western blot analysis

Western blotting was performed as described previously^25^. Frozen SCN tissue samples were homogenized by sonication in ice-cold extraction buffer (150 mM NaCl, 50 mM Tris HCl, 1 mM EDTA, 1% Triton X-100, 1% protease inhibitor cocktail; P8340, Sigma-Aldrich, St Louis, MO, USA) and the protein concentration was then determined by a Bio-Rad DC protein assay kit (500-0116, Bio-Rad, Hercules, CA, USA). The proteins (20 µg) were electrophoresed on 7.5% acrylamide gel and then electrotransferred to PVDF membrane (GE healthcare Biosciences, Piscataway, NJ, USA). Membranes were blocked for 1 hr at room temperature with 5% non-fat milk in Tris-buffered saline containing 0.1% Tween 20 (TBST) and then incubated overnight at 4 °C with primary antibody against NHE1 (rabbit anti-NHE1; 1:5000; ab67314, RRID:AB_1141782; Abcam, Cambridge, MA, USA). After washing with TBST, membranes were processed with horseradish peroxidase-conjugated anti-rabbit secondary antibody (1:5000), and the protein bands were visualized using chemiluminescence (ECL reagent; GE healthcare Biosciences, Piscataway, NJ, USA). After detection of NHE1-immunoreactive bands, the same membranes were stripped and reprobed with β-actin monoclonal antibody (1:20000; A5441, RRID:AB_476744; Sigma-Aldrich, St Louis, MO, USA) to confirm equal protein loading. For each sample, the optical density of the NHE1 band was quantified with ImageJ 1.45 s (NIH), normalized to the loading control (β-actin), and then averaged across all gels.

### Experimental design and statistical analyses

Lights-on was designated Zeitgeber time (ZT) 0. For comparing the data recorded between day (ZT 4–11) and night (ZT 13–20), the animal was killed at ZT 2 and ZT 10, respectively.

Data were analyzed and plotted with custom-made programs written in Visual Basic 6.0. Statistical analyses were performed with the commercial software GraphPad PRISM (RRID:SCR_002798; GraphPad Software, San Diego, CA, USA). Data were given as means ± SEM. Statistical comparisons of means between two groups were analyzed using the unpaired (Figs 2D and 3D) or paired (Figs 8D2,E2, 9B,C, 10D,E) two-tailed Student’s *t* test. Multiple comparisons among means were analyzed with one-way ANOVA, followed by *post hoc* Tukey’s test for selected pairs comparison (Figs 2B and 5A,B). A *p-*value of less than 0.05 is considered statistically significant.

## Supplementary information

Supplementary information
