# Supplementary material for: The Na+/H+-Exchanger NHE1 Regulates Extra- and Intracellular pH and Nimodipine-sensitive [Ca2+]i in the Suprachiasmatic Nucleus
Source: Sci Rep. 2019 Apr 23;9:6430. doi: 10.1038/s41598-019-42872-w (PMC6478949; doi:10.1038/s41598-019-42872-w)
Supplement: Supplementary file 1 — Supplementary information [file 41598_2019_42872_MOESM1_ESM.pdf]

The Na^+/H^+-Exchanger NHE1 Regulates Extra- and Intracellular pH and Nimodipine-sensitive $[Ca^{2+}]_i$ in the Suprachiasmatic Nucleus

Pi-Cheng Cheng, Hsin-Yi Lin, Ya-Shuan Chen, Ruo-Ciao Cheng, Hung-Che Su, and Rong-Chi Huang

Supplementary data

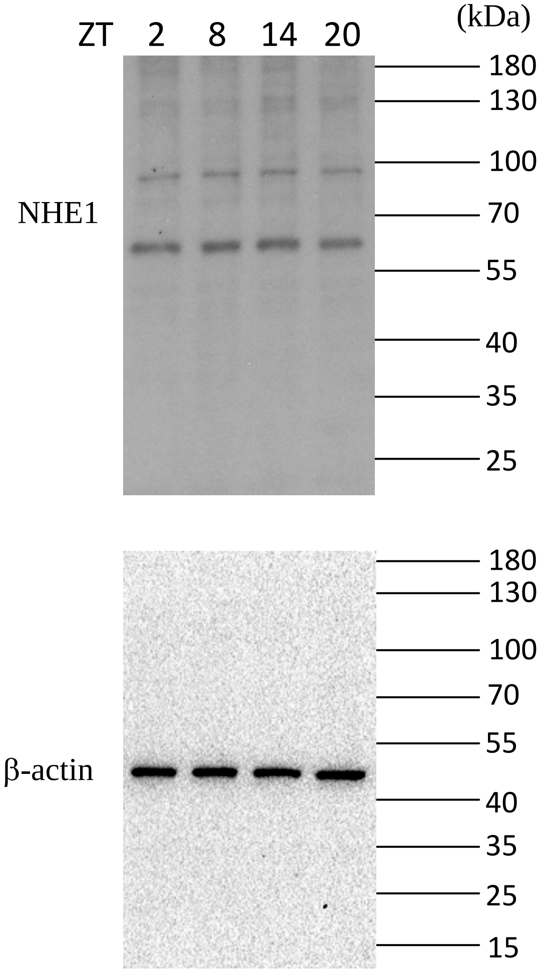

We consistently observed two immunoreactive bands on our Western blots: a larger band of ~90 kDa that represents fully glycosylated NHE1 and a smaller band that represents unprocessed or only partially glycosylated protein

The raw data of the band between 55-70 kDa				
	ZT 2	ZT 8	ZT 14	ZT 20
Exp. 1	1.226797824	1.33033108	1	1.154384539
Exp. 2	1.173480257	1.241838582	1.078021874	1
Exp. 3	1.370436493	1	1.234875657	1.267231541
Exp. 4	1.039483681	1.255804159	1.231532218	1

The western blot analysis also revealed no day-night variation in the NHE1 protein levels.
